# Salt and potassium intake among adult Ghanaians: WHO-SAGE Ghana Wave 3

**DOI:** 10.1186/s40795-020-00379-y

**Published:** 2020-09-29

**Authors:** Elias K. Menyanu, Barbara Corso, Nadia Minicuci, Ilaria Rocco, Joanna Russell, Lisa J. Ware, Richard Biritwum, Paul Kowal, Aletta E. Schutte, Karen E. Charlton

**Affiliations:** 1 School of Medicine, Faculty of Science, Medicine and Health, University of Wollongong, Northfields Avenue, Wollongong, NSW 2522 Australia; 2 Neuroscience Institute, National Research Council, Padova, Italy; 3 School of Health and Society, Faculty of Social Sciences, University of Wollongong, Northfields Avenue, Wollongong, NSW 2522 Australia; 4 SAMRC/Wits Developmental Pathways for Health Research Unit, University of the Witwatersrand, Johannesburg, South Africa; 5 DSI-NRF Centre of Excellence in Human Development, University of the Witwatersrand, Johannesburg, South Africa; 6 Department of Community Health, University of Ghana, Accra, Ghana; 7 Chiang Mai University Research Institute for Health Sciences, Chiang Mai, Thailand; 8 World Health Organization (WHO), Geneva, Switzerland; 9 School of Public Health and Community Medicine, University of New South Wales; The George Institute for Global Health, Sydney, NSW 2052 Australia; 10 Hypertension in Africa Research Team, North-West University, Potchefstroom, 2520 South Africa; 11 Illawarra Health and Medical Research Institute, Wollongong, NSW 2522 Australia

**Keywords:** Hypertension, Salt intake, Potassium, 24 h urine, Ghana

## Abstract

Though Ghana has high hypertension prevalence, the country lacks current national salt consumption data required to build and enhance advocacy for salt reduction. We explored the characteristics of a randomly selected sub sample that had valid urine collection, along with matched survey, anthropometric and BP data (*n* = 839, mean age = 60y), from the World Health Organization’s Study on global AGEing and adult health (WHO-SAGE), Ghana Wave 3, *n* = 3053). We also investigated the relationship between salt intake and blood pressure (BP) among the cohort. BP was measured in triplicate and 24 h urine was collected for the determination of urinary sodium (Na), potassium (K), creatinine (Cr) and iodine levels. Hypertension prevalence was 44.3%. Median salt intake was 8.3 g/day, higher in women compared to men (8.6, interquartile range (IQR) 7.5 g/day vs 7.5, IQR 7.4 g/day, *p* <  0.01), younger participants (18–49 y) compared to older ones (50+ y) (9.7, IQR 7.9 g/day vs 8.1, IQR 7.1 g/day, *p* <  0.01) and those with higher Body Mass Index (BMI) (> 30 kg/m^2^) compared to a healthy BMI (18.5–24.9 kg/m^2^) (10.04, IQR 5.1 g/day vs 6.2, IQR 5.6 g/day, *p* <  0.01). More than three quarters (77%, *n* = 647) of participants had salt intakes above the WHO maximum recommendation of 5 g/d, and nearly two thirds (65%, *n* = 548) had daily K intakes below the recommended level of 90 mmol. Dietary sodium to potassium (Na: K) ratios above 2 mmol/mmol were positively associated with increasing BP with age. Population-based interventions to reduce salt intake and increase K consumption are needed.

## Introduction

Cardiovascular diseases (CVDs) remain a major contributory factor for mortality and morbidity worldwide, accounting for 17.9 million deaths in 2016, and representing a third of all deaths globally [1]. While Low- and Middle-Income Countries (LMICs) already share the greatest impact of CVD-related deaths, with rates of 300–600 deaths per 100,000 population, these are predicted to increase, causing premature, avoidable loss of lives [2]. Hypertension is a major risk factor for CVD and accounted for more than 50% of CVD-specific deaths in 2012 [3]. It has been estimated that the burden of hypertension will increase to approximately 1.56 billion people globally by 2025, with larger populations of hypertensives living in LMICs [3–5].

Hypertension is a common condition in Ghana, where its prevalence has increased more than two-fold over just two decades (1988–2007) [6]. A recent nationally representative study among participants largely 50 years and older, in Ghana, reported the prevalence of hypertension to be 58.9% in adults with about one-fifth of those with hypertension being aware of having the condition [7]]. Reducing population-level prevalence of hypertension by 30% by the year 2030 is identified by WHO as one of the nine voluntary global targets to reduce non communicable diseases (NCDs) [8]. Salt reduction has been identified by the WHO as one ‘best buys’ approach to reduce overall CVD risk through lowering of (BP) [9].

Increased consumption of salt (> 2 g Na/day, equivalent to 5 g salt/day) together with insufficient potassium (K) intake (< 3.5 g/day) are strongly associated with a number of NCDs including hypertension [10–14]. Several systematic literature reviews, meta analyses and randomized-controlled trials have shown that reduced sodium (Na) consumption results in a decline in blood pressure in both hypertensives and normotensive adults [12, 14–17], which saves lives [18]. In view of this, ‘WHO’s SHAKE the Salt Habit’ resource has emphasized the importance of measuring and monitoring population salt consumption patterns in order to inform stakeholders about strategies to reduce salt intake and evaluate the effect of any implemented salt reduction programmes [19]. Though Ghana is committed to the World Health Assembly’s target of 30% relative reduction in the population salt intake by 2025 [10], the country has made slow progress towards achieving the target due to other competing public health priorities and limited resources [20].

Few studies have been conducted to assess salt intake in the Ghanaian population [21]. A lack of recent, nationally representative data on population salt consumption among adults has further been a disincentive for advocacy around salt reduction efforts [22]. The current study was undertaken to obtain a reliable estimate of population salt intake and salt use behaviour, and to assess the relationship between salt and K intake with blood pressure (BP) in Ghanaian adults.

## Methods

### Study design, population and outcomes

**Fig. 1:**
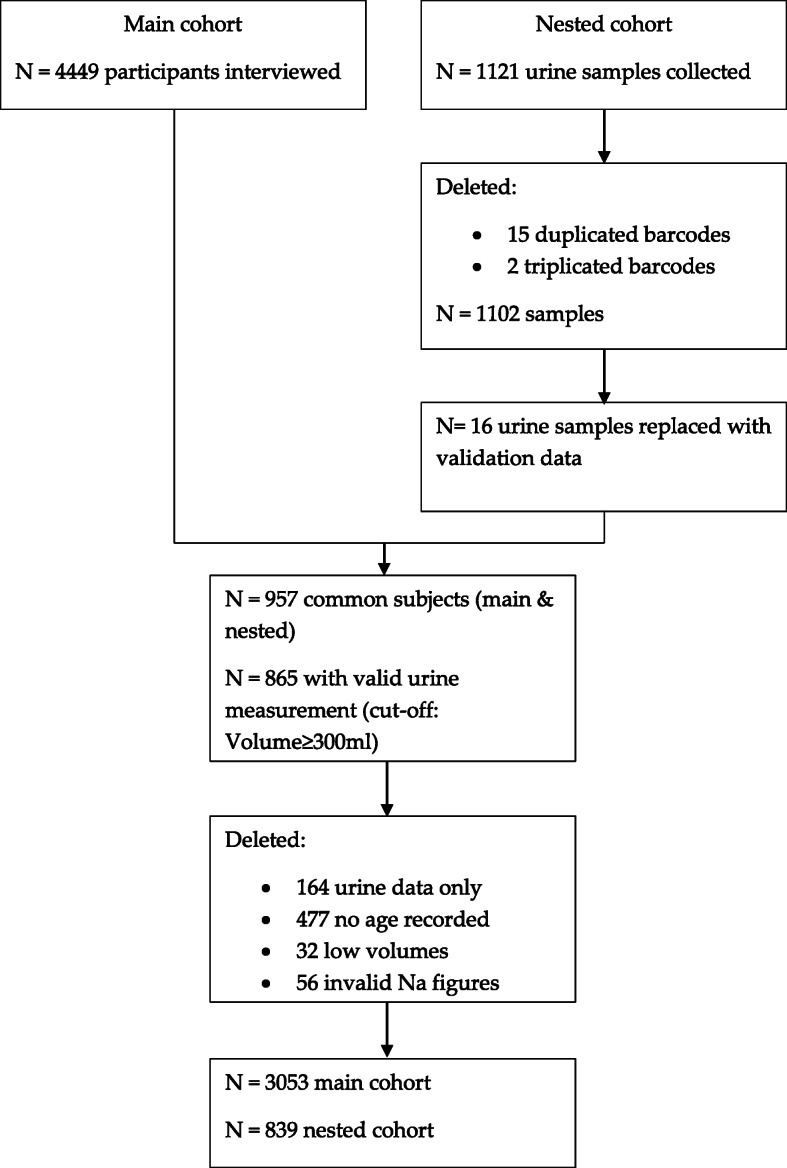
Study flow chart

This study is a nested sub study of WHO’s Study on global AGEing and adult health (WHO - SAGE) Wave 3 Ghana, which collected 24 h urine samples for analysis of Na and K [23]. WHO - SAGE is a longitudinal cohort study conducted in six LMICs (China, Ghana, India, Russia, Mexico and South Africa) with the aim to examine the ageing process and address health inequalities among adult populations [24]. Participants were sampled based on a design used in the 2003 World Health Survey with primary sampling units (households) stratified by region and location (urban/rural) The study design randomly selects 24 households from an enumeration area (twenty 50+ year households and four 18-49 year households). In the 50+ year households, all participants are selected and compares with a smaller number of participants selected from the 18-49 year households, details of which are described elsewhere [25]. WHO SAGE Ghana Wave 3 data collection was completed over a period of 1 year (July 2018–June 2019) with *n* = 1100 24 h urine samples collected nationwide. Out of 5570 participants recruited for WHO - SAGE Ghana Wave 3, 1102 (20%), were randomly selected to provide urine samples (nested sub study). Of these, 76.7% (*n* = 844) had successful (BP) data and 88.6% (*n* = 976) had complete urine samples. However, fewer participants (*n* = 839) had a complete urine collection, accompanied with survey, anthropometric and valid BP data (Fig. 1).

Field worker teams consisted of 3–5 interviewers per team who visited participants in their homes and workplaces to conduct interviews. All field workers were trained by the central WHO SAGE team with standardized survey materials [24]. Surveys were conducted in the participants’ preferred home language with the use of the computer assisted personal interview (CAPI) method.

The WHO/Pan American Health Organization (PAHO) protocol was utilised in the determination and collection of Na, K and Cr in the 24 h urine collections. In collecting urine samples, 5-l urine bottles containing 1 g thymol as preservative, were given to participants to collect 24 h urine. The collection procedure was thoroughly explained to participants. In brief it is as follows: 1) void the ‘first urine’ and note the time but include the ‘last urine’ 2) keep the bottles to yourself and collect only your own urine 3) collect all urine passed within the 24 h and 4) keep collected urine in a cool place [26]. The 24 h urine was collected, thoroughly mixed, volumes recorded, 3 aliquots of 5 ml were generated and samples kept in cold boxes and transported to the Noguchi Memorial Institute for Medical Research of the University of the Ghana (NMIMR-UG). Urine samples were considered valid if the volume was ≥300 ml [27, 28]. Additionally, iodine was assessed for analysis because Ghana had a universal iodine programme in place.

Analysis were undertaken by NMIMR-UG, that followed the WHO/PAHO protocol for quantitative analysis of Na, K, Cr in urine samples [26]. Urine samples for iodine analyses were stored at − 20° and batch analysed using the Sandell-Kolthoff method with ammonium persulfate digestion and microplate [29]. Creatinine excretion in this population was much lower than that reported for many other populations, from which Cr cut-off values have been determined to indicate completeness of 24 h urine collection [28]. Because of this, only urine volume was used to indicate whether urine samples were considered to represent completeness. This approach has been used in other multi-country studies including; the International Population Study on Macronutrients and Blood Pressure [INTERMAP] and the International Study of Sodium, Potassium, and Blood Pressure [INTERSALT] [27]. In the first enumerator areas (EAs) sampled within the Accra region, some calculated salt values were unfeasibly high (> 40 g/d, *n* = 40,) due to unacceptable storage conditions [30]. A follow-up validation study conducted in *n* = 67 participants included the collection of repeated 24 h urine samples from the same participants in one EA (Alajo; *n* = 19), while an additional two non-urine EAs from the larger survey sample were visited as a comparator (Mataheko; *n* = 24 and Nima; *n* = 24). A second laboratory (Liberty Medical Laboratory, Accra) also performed duplicate analyses in the validation study. Values for Dosise EA (*n* = 23) were excluded after the validation study because they were confirmed as being unfeasible, and the validation study values replaced original measures for the case of Alajo (*n* = 16; 3 barcodes of urine samples in validation study were excluded because they could not be matched with those of the original study) (Supplementary Table 1).

Sodium (mmol/l) in the 24 h urine sample was converted to salt (g/d) using the formula: Na mmol/l × 24 h volume (litres) × 23.1 (molecular weight of Na)/390 (390 mg Na per 1 g Nacl (salt). From this variable, participants were categorised as having low (< 5 g/day), medium (5–9 g/day) or high (> 9 g/day) salt intake. K (mmol/l) in the 24 h urine sample was converted to K (mmol/d) using the formula: K (mmol/l) × 24 h volume (litres) [31]. The ratio Na (mmol/L) to K (mmol/L) was created (Na:K) and categorised as low (< 2), medium (2–5) or high (> 5). BP was measured by field workers with wrist worn BP devices with positional sensors (Omron R6, Japan) [32] that have been validated by the European Society of Hypertension International Protocol [32–34]. After being seated with legs uncrossed for 5 min, three BP readings were taken on the left wrist (1 min between each measurement) while the wrist was placed precisely at the level of heart. The last two readings were averaged as a measure of the participant’s BP. Hypertension classification was based on the European Society for Hypertension Guidelines (2018) which defines hypertension as systolic ≥140 and/or diastolic ≥90 mmHg [35]. BP readings in the database were determined valid if: Systolic (SBP) > Diastolic (DBP); and SBP was between 80 and 270 mmHg; and DBP was between 40 and 180 mmHg; and SBP minus DBP (Pulse Pressure, PP) > 13 mmHg. Mean Arterial Pressure (MAP) is the average arterial pressure throughout one cardiac cycle, systole, and diastole. MAP was calculated using the formulae: MAP = SBP + 2 (DBP)/3 [36]. Hypertension status was measured as self-reported treatment or having a measured BP ≥ 140/90 mmHg. Hypertension awareness was based on self-reported previous diagnosis of hypertension in those with BP ≥ 140/90 mmHg. Treatment was determined from self-reported medication use for hypertension within the last 2 weeks. Hypertension control was determined by self-reported medication use within the last 2 weeks and a measured BP less than 140/90 mmHg. Individuals with no current medication use and a measured BP < 140/90 mmHg were categorized as non-hypertensive.

Weight was measured with calibrated scales, while height was measured with a portable stadiometer. Data were also collected on health indicators such as tobacco, alcohol use, salt use behaviours and previous disease conditions. Physical activity levels were measured using the Global Physical Activity Questionnaire [37]. For salt use behaviour questions, responses such as ‘always’ and ‘often’ were combined to represent ‘frequent use’, whilst ‘sometimes’ and ‘rarely’ were combined to represent ‘infrequent use’.

Prior to taking part in the study, study measures were explained to participants in their home languages and written informed consent was obtained. The study complied with the Declaration of Helsinki with ethical approval from the WHO Ethics Committee (RPC 149) and the University of Ghana Medical School Ethics and Protocol Review Committee (MS-Et/M.03 - P 3.1/2005–2006).

### Statistical analysis

Data were analysed using Stata Statistical Software: Release 15 (Stata Corp LLC, 2017; College Station, USA). Data distribution was checked visually and with the Shapiro Wilks test for normality. Descriptive statistics of frequencies, percentages and median (interquartile range, IQR) were used to describe participants’ characteristics. Categorical variables were evaluated using frequencies, Pearson’s Chi-Square and Fisher’s Exact tests. Comparison between groups for non-parametric data was examined using Mann-Whitney U and Kruskal-Wallis tests and Spearman’s Rho was used to test for correlations. Linear regressions were used to evaluate the associations between variables. To test regression slopes for equality between the three salt level groups and between the three Na:K ratio groups, the interaction terms of groups with age was added into the models. To test if a difference in the group’s coefficients (i.e. slope) was present, the F test was applied. If significant, post-estimation was pursued to identify which group differed from the others.

## Results

Table 1 compares sociodemographic characteristics of the nested sub study sample with those included in the total Wave 3 survey collection.

**Table 1:** Demographic profile of SAGE GHA W3 survey participants and subsample with valid urine data (urine data considered valid if volume ≥ 300 ml)

	*Main SAGE cohort (n = 3053)*	*Salt sub study sample (n = 839)*	*P-value*
*Age*			
Years	60 (20)	60 (19)	0.3294
18–49 years, n (%)	717 (23.5)	174 (20.7)	0.0100
50 plus years, n (%)	2336 (76.5)	665 (79.3)	0.4494
*Sex, n (%)*			0.001
Men	1183 (38.8)	371 (32.3)	
Women	1870 (61.2)	568 (67.7)	
*Ethnicity, n (%)*			< 0.0001
Akan	1544 (50.6)	497 (59.2)	
Ewe	216 (7.1)	58 (6.9)	
Ga-Adangbe	224 (7.3)	45 (5.4)	
Gruma	30 (1.0)	6 (0.7)	
Grusi	76 (2.5)	12 (1.4)	
Guan	38 (1.2)	16 (1.9)	
Mande-Busanga	63 (2.1)	3 (0.4)	
Mole-Dagbon	174 (5.7)	24 (2.9)	
Other	612 (20.0)	174 (20.7)	
*Residence, n (%)*			< 0.0001
Urban	1301 (42.6)	457 (54.5)	
Rural	1752 (57.4)	382 (45.5)	
*Marital status, n (%)*			0.002
Never married	208 (6.8)	39 (4.6)	
Married/cohabiting	1760 (57.7)	470 (56.0)	
Separate/divorced	369 (12.1)	87 (10.4)	
Widowed	715 (23.4)	243 (29.0)	
*Never been to school, n (%)*	1119 (36.7)	240 (28.6)	< 0.0001
*Education (years)*	10 (5)	10 (5)	0.0139
*BMI kg/m*^*2*^	24.2 (6.8)	25.4 (7.4)	< 0.01
*Waist to height ratio*	0.54 (0.11)	0.56 (0.13)	< 0.01
*Never used alcohol, n (%)*	1865 (61.3)	483 (57.6)	0.052
*Never used tobacco, n (%)*	2739 (90.0)	759 (90.5)	0.68
*Systolic BP (mm Hg)*	124 (26.5)	126.5 (29)	< 0.01
*Diastolic BP (mm Hg)*	76 (17)	76.5 (17)	0.83
*Mean Arterial Pressure (Map) mmHG*	92.5 (19.2)	93.2 (20.7)	0.24
*Hypertension, n(%)*	1132 (37.6)	370 (44.3)	< 0.01
*Diabetes, n(%)*	157 (5.2)	67 (8.0)	0.01
*Portions of fruit, n(%)*			0.05
*Met WHO’s recommendation of at least 5 servings of friuts and vegetables*	1591 (57.4)	442 (54.5)	0.14
*Frequently add salt to food at table, n(%)*	642 (21.1)	107 (12.7)	< 0.01
*Frequently add salt to food during cooking, n(%)*	2527 (83.0)	672 (80.1)	0.05
*Believe they consume too much salt, n(%)*	265 (8.8)	92 (11.0)	0.05
*Believe a high salt diet is bad for health, n(%)*	2247 (75.9)	651 (80.0)	0.02
*Regularly control of salt intake, n(%)*	1243 (42.1)	394 (48.5)	< 0.01

Nested sub sample: all respondent with CAPI data and valid urine, sex and age recorded. Some variables may contain missing data. Data are presented as median (IRQ) unless otherwise indicated. Hypertensive by measured BP ≥ 140 and/or 90 mmHg or previous diagnosis (self-reported). Ethnicity, marital status, education, alcohol/tobacco use and diabetes prevalence by self-report. Mann-Whitney Test used to compare medians, Pearson Chi-Square test and Fisher’s Exact Test used to compare proportional data

Hypertension prevalence in the nested sub study was 44.3% (*n* = 370) with 41.4 and 46.1% for men and women respectively. Of those with hypertension, 59.7% were aware of their condition (*n* = 222) of which 69.1% (*n* = 154) were receiving prescribed antihypertensive medication. Of those being treated for hypertension, 51% (*n* = 77) had controlled BP (ie BP ≤ 140/90).

Among participants with both urine, survey data and BP data (*n* = 839), median intake of salt was equivalent to 8.3 g/day (IQR 7.5) (mean = 10.2 g ± 7.2). Salt intake was higher in: (1) women compared to men; median 8.6 (7.5)g/day vs 7.5 (7.4)g/day, *p* <  0.01; (2) younger participants (18–49 y) compared to older ones (50+ y); median 9.7 (7.9)g/day vs 8.1 (7.1)g/day, *p* <  0.01; and (3) those with higher BMI (> 30 kg/m^2^) compared to a desirable BMI (18.5–24.9 kg/m^2^); median 10.04 (5.1)g/day vs 6.2 (5.6)g/day, *p* <  0.01. There was no significant difference in salt consumption between rural and urban dwellers. Overall, 77.7% (*n* = 647) of participants had salt intakes above the WHO maximum recommendation of 5 g/d, with 38.9% consuming more than twice this level (> 10 g/d, *n* = 321) and 16.3% consuming more than three times the recommended level (> 15 g/d, *n* = 137). More men than women (27.1% (*n* = 73) vs 19.9% (*n* = 122); *p* = 0.02) and older participants than younger ones (24.8% (*n* = 164) vs 12.1% (*n* = 21); *p* <  0.01) achieved the WHO’ salt recommendation of ≤5 g salt/day (Supplementary Tables 2 and 3).

According to groupings by salt intake (low, < 5 g/d; medium, 5-9 g/d; high, > 9 g/d), those with higher salt intakes were significantly younger, had higher BMI, a higher urinary K excretion, a higher urinary Na: K and lower urinary iodine concentrations. However, BP and hypertension status did not differ between the groups (Table 2).

**Table 2:** Nested cohort by salt excretion levels, low, medium and high salt groups, WHO – SAGE Ghana Wave 3 (2019)

Characteristics	Low salt < 5 g/d (*n* = 186)	Medium salt 5-9 g/d (*n* = 269)	High salt > 9 g/d (*n* = 378)	*p* value
*Age yrs*	64 (20)	60 (17)	58 (18)	< 0.01
*Sex male, n (%)*	*n* = 185 73 (39.5)	*n* = 269 90 (33.5)	*n* = 378 105 (27.8)	0.02
*Ethnicity n (%)*	*n* = 184	*n* = 268	*n* = 376	0.03
Akan	122 (66.3)	168 (62.7)	203 (54.0)	
Ga	7 (3.8)	11 (4.1)	27 (7.2)	
Ewe	14 (7.6)	18 (6.7)	24 (6.4)	
Mole-dagbon	5 (2.7)	6 (2.2)	13 (3.5)	
Mole-busanga	1 (0.5)	0 (0.0)	2 (0.5)	
Grusi	3 (1.6)	3 (1.1)	6 (1.6)	
Guan	0 (0.0%)	11 (4.1%)	5 (1.3)	
Gruma	1 (0.5)	3 (1.1)	2 (0.5)	
Other	31 (16.8)	48 (17.9)	94 (25.0)	
*Urban n (%)*	*n* = 185 102 (55.1)	*n* = 269 149 (55.4)	*n* = 378 200 (52.9)	0.79
*Education (years)*	*n* = 118 10 (5)	*n* = 179 10 (3)	*n* = 250 10 (5)	0.07
*BMI, kg/m*^*2*^	*n* = 179 23.8 (6.5)^a^	*n* = 258 25.5 (7.8)	*n* = 356 26.2 (7.5)^a^	< 0.01
*Salt intake g/d*	*n* = 186 3.6 (1.5)^ab^	*n* = 269 6.7 (2.0)^ac^	*n* = 371 13.4 (8.9)^bc^	< 0.01
*K intake, mmol/d*	*n* = 186 32.0 (25.8)^ab^	*n* = 269 54.9 (43.8)^ac^	*n* = 378 112.3 (115.8)^bc^	< 0.01
*Na: K*	*n* = 186 1.7 (1.2)^ab^	*n* = 269 2.0 (1.4)^ac^	*n* = 378 2.3 (1.9)^bc^	< 0.01
*Urinary iodine excretion, mmol/day*	*n* = 150 166.2 (168.5)^b^	*n* = 211 148.2 (131.4)^a^	*n* = 292 117.6 (132.8)^ab^	< 0.01
*Systolic BP, mmHg*	*n* = 186 129 (31.0)	*n* = 266 126 (31.0)	*n* = 375 126.5 (29.0)	0.73
*Diastolic BP, mmHg*	*n* = 188 76 (18.0)	*n* = 270 75.5 (17)	*n* = 379 77.5 (117.0)	0.68
*Pulse pressure, mmHg*	*n* = 186 50.5 (16.7)	*n* = 266 49.5 (17.6)	*n* = 375 49.5 (16)	0.20
*Mean Arterial Pressure (MAP), mmHg*	*n* = 185 94.2 (21.3)	*n* = 266 110 (41.0)	*n* = 376 93.5 (19.4)	0.85
*Hypertension* Prevalence, n (%)	*n* = 186 94 (50.5)	*n* = 268 110 (41.0)	*n* = 378 166 (44.3)	0.13
Hypertension awareness, n (% of hypertension prevalence)	*n* = 94 51 (54.8)	*n* = 110 62 (55.4)	*n* = 166 108 (65.1)	0.16
Hypertension medication, current use n (% of hypertension awareness)	*n* = 51 31 (60.8)	*n* = 62 47 (75.8)	*n* = 108 74 (68.5)	0.23
Hypertension control, n (% of Hypertension medication)	*n* = 31 16 (51.6)	*n* = 47 22 (46.8)	*n* = 74 39 (52.7)	0.82
*Stroke, n (%)*	*n* = 185 6 (3.2)	*n* = 269 6 (2.2)	*n* = 378 6 (1.6)	0.44
*Diabetes, n (%)*	*n* = 185 10 (5.4)	*n* = 269 24 (8.9)	*n* = 378 32 (8.5)	0.37
*Tobacco use, n (%)*	*n* = 182 25 (13.7)	*n* = 269 25 (9.3)	*n* = 378 29 (7.7)	0.72
*Alcohol use, n (%)*	*n* = 182 80 (44.0)	*n* = 269 115 (42.8)	*n* = 378 156 (41.3)	0.82
*Fruits and Veg., met recommendation n (%)*	*n* = 186 97 (52.2)	*n* = 269 152 (56.5)	*n* = 378 193 (51.1)	0.38
*Frequently adds salt to food at the table, n (%)*	*n* = 158 24 (13)	*n* = 269 35 (13)	*n* = 279 (12.4)	0.65
*Frequently adds salt to cooking at home, n (%)*	*n* = 185 149 (80.5)	*n* = 296 225 (83.6)	*n* = 378 291 (77)	0.05
*Believe that they are eating just the right amount of salt n (%)*	*n* = 185 105 (56.8)	*n* = 267 169 (63.3)	*n* = 376 228 (60.6)	0.18
*Believe a high salt diet can cause a serious health problem, n (%)*	*n* = 180 144 (80.0)	*n* = 259 207 (79.9)	*n* = 368 296 (80.4)	0.99
*Does something to control salt consumption, n (%)*	*n* = 183 82 (44.8)	*n* = 260 126 (48.5)	*n* = 363 182 (50.1)	0.50

Data are presented as median (IQR, interquartile range) unless otherwise stated. BMI, body; hypertension prevalence, BP ≥ 140/90 or previous diagnosis; Current use of hypertension medication represents current use in the last 2 weeks; BMI, body mass index; tobacco use by self-report; alcohol use by self-report; met fruits and vegetable recommendation indicates consumed five or more servings of fruits and vegetables a day; salt behaviour responses, frequently indicates ‘always’ and ‘often’; continuous variables compared using Independent Kruskal-Wallis Test and categorical variables compared using Pearson Chi-Square Test and Fisher’s Exact Test. a, b, c; sig at *p* < 0.05

In response to the salt behaviour questions (Supplementary Table 4), 12.8% (*n* = 107) of participants reported that they frequently (always and often) added salt to food at the table with significantly greater numbers of younger (27.3%, *n* = 28, *p* = 0.02) and rural (61%, *n* = 61, < 0.01) dwellers responding affirmatively to this question. More than three quarters (80.1%, *n* = 671) of the surveyed population reported that they frequently added salt to food at home during cooking while 60.9% (*n* = 508) perceived themselves to consume the right amount of salt. While 19.9% (*n* = 126) did not know that a high salt diet could cause a serious health problem, 48.5%, (*n* = 394) of the participants reported actively doing something on a regular basis to control their salt intake. Of those who knew a high salt diet could cause a serious health problem, 58.9% (*n* = 378) did something on a regular basis to control their salt intake.

Almost two-thirds (65%, *n* = 548) of participants did not meet the daily K recommendation of ≥90 mmol [38], with those in the lowest salt intake group having the lowest urinary concentrations. While 42.6% (*n* = 141) of urban participants met the guidelines for K intake, this was the case for only 28.6% (*n* = 101) of participants residing in rural areas (*p* <  0.01). Only 7.6% (*n* = 63) achieved the recommended Na: K ratio of ≤1 mmol [38]. There was a strong positive correlation between 24 h urinary Na and K concentrations (Spearman’s rho = 0.71, *p* <  0.01). Linear regression indicated that urinary Na excretion accounted for more than half the variability in urinary K excretion (*R*^2^ = 0.51, F (1, 832) = 861, *p* = < 0.01). Urinary K excretion increased by 0.71 mmol/day for each mmol/day increase in urinary Na such that Na: K ratio was significantly higher in the high salt group compared to the low salt group (median 2.3 (1.9)g/day vs 1.7 (1.9)g/day, respectively, *p* <  0.01).

The high Na: K group had a significantly higher BMI than those in the lowest Na: K group (median 28.1, IQR 8.2 vs 24.8, IQR 7.0, *p* = 0.01) and had higher number of participants who reported doing something on regular basis to control their salt intake than those in the lowest Na: K group (61.5%, *n* = 15 vs 42.7%, *n* = 166, *p* <  0.01) (Table 3).

**Table 3:** Nested cohort by urinary sodium to potassium (Na:K) ratio, low, medium and high groups, WHO – SAGE Ghana Wave 3 (2019)

Characteristics	Low Na: K (< 2) *n* = 399	Medium Na: K (2–5) *n* = 389	High Na: K (> 5) *n* = 41	*p* value
*Age yrs*	60 (18)	61 (19)	58 (22)	0.44
*Sex male, n (%)*	*n* = 399 139 (34.8)	*n* = 389 116 (29.8)	*n* = 41 12 (29.3)	0.29
*Ethnicity n (%)*	*n* = 397	*n* = 387	*n* = 41	< 0.01
Akan	229 (57.7)	246 (63.6)	17 (41.5)	
Ga	5 (1.3)	29 (7.5)	10 (24.4)	
Ewe	31 (7.8)	23 (5.9)	1 (2.4)	
Mole-dagbon	13 (3.3)	11 (2.8)	0 (0.0)	
Mole-busanga	2 (0.5)	1 (0.3)	0 (0.0)	
Grusi	7 (3.0)	5 (1.3)	0 (0.0)	
Guan	12 (3.0)	4 (1.0)	0 (0.0)	
Gruma	3 (0.8)	3 (0.8)	0 (0.0)	
Other	95 (23.9)	65 (16.8)	173 (21)	
*Urban n (%)*	*n* = 398 183 (46.0)	*n* = 389 240 (61.7)	*n* = 41 26 (63.4)	< 0.01
*Education (years)*	*n* = 253 10 (4)	*n* = 267 10 (5)	*n* = 26 10 (6)	0.51
*BMI kg/m*^*2*^	*n* = 387 24.8 (7.0)	*n* = 364 25.7 (7.4)	*n* = 39 28.1 (8.2)	0.04
*Salt intake g/d*	*n* = 398 7.5 (7.4)^ab^	*n* = 384 8.6 (6.6)^ac^	*n* = 40 13.9 (11.9)^bc^	< 0.01
*K intake, mmol/d*	*n* = 399 93.4 (97.8)^ab^	*n* = 389 49.5 (38.7)^b^	*n* = 41 38.2 (38.4)^a^	< 0.01
*Na: K*	*n* = 399 1.4 (0.6)^ab^	*n* = 389 3.0 (1.3)^ac^	*n* = 41 5.5 (1.3)^bc^	< 0.01
*Urinary iodine excretion, mmol/day*	*n* = 383 129.7 (116.0)^a^	*n* = 370 153.9 (164.4)	*n* = 40 146.9 (193.9)^a^	< 0.01
*Systolic BP, mmHg*	*n* = 398 126 (26)	*n* = 385 126 (32.0)	*n* = 40 125 (34.0)	0.03
*Diastolic BP, mmHg*	*n* = 398 77 (17.0)	*n* = 385 75 (17.0)	*n* = 40 77 (18.0)	0.25
*Pulse pressure, mmHg*	*n* = 398 48 (16.6)	*n* = 385 51 (17.0)	*n* = 40 51.7 (15.0)	0.26
*Mean Arterial Pressure (MAP), mmHg*	*n* = 399 93.7 (18.8)	*n* = 387 92.5 (21.2)	*n* = 41 91.7 (25.3)	0.61
*Hypertension* Prevalence, n (%)	*n* = 398 170 (42.7)	*n* = 387 178 (46.0)	*n* = 40 18 (45.0)	0.65
Hypertension awareness, n (% of hypertension prevalence)	*n* = 170 102 (60.0)	*n* = 178 106 (59.6)	*n* = 18 11 (61.1)	0.10
AHT, current use n (% of hypertension awareness)	*n* = 102 65 (63.7)	*n* = 106 79 (74.8)	*n* = 11 7 (63.6)	0.22
Hypertension control, n (% of AHT)	*n* = 63 33 (52.5)	*n* = 78 40 (51.3)	*n* = 7 3 (42.9)	0.89
Stroke, n (%)	*n* = 399 11 (2.8)	*n* = 389 7 (1.8)	*n* = 41 0 (0.0)	0.45
*Diabetes, n (%)*	*n* = 399 34 (8.5)	*n* = 389 30 (7.7)	*n* = 41 2 (4.9)	0.69
*Tobacco use, n (%)*	*n* = 399 45 (11.3)	*n* = 389 34 (8.7)	*n* = 41 0 (0.0)	0.05
*Alcohol use, n (%)*	*n* = 399 160 (40.1)	*n* = 389 172 (44.2)	*n* = 41 19 (46.3)	0.44
*Fruits and Veg., met recommendation n (%)*	*n* = 399 212 (53.1)	*n* = 389 203 (52.2)	*n* = 41 24 (58.5)	0.74
*Frequently add salt to food at the table, n (%)*	*n* = 399 52 (13)	*n* = 389 48 (12.3)	*n* = 41 6 (14.6)	0.88
*Frequently add salt to cooking at home, n (%)*	*n* = 399 320 (80.0)	*n* = 389 311 (79.9)	*n* = 41 33 (80.5)	0.30
*Believes that they are eating just the right amount of salt n (%)*	*n* = 397 247 (62.2)	*n* = 387 231 (59.7)	*n* = 41 24 (58.5)	0.48
*Believes a high salt diet can cause a serious health problem, n (%)*	*n* = 389 304 (78.1)	*n* = 375 311 (82.9)	*n* = 40 30 (75.0)	0.18
*Does something to control salt consumption, n (%)*	*n* = 389 166 (42.7)	*n* = 375 209 (55.7)	*n* = 39 15 (61.5)	< 0.01

Data are presented as median (IQR, interquartile range) unless otherwise stated. BMI, body; hypertension prevalence, BP ≥ 140/90 or previous diagnosis; AHT, antihypertensive medication use in the last 2 weeks; BMI, body mass index; tobacco use by self-report; alcohol use by self-report; met fruits and vegetable recommendation indicates consumed five or more servings of fruits and vegetables a day; salt behaviour responses, frequently indicates ‘always’ and ‘often’; continuous variables compared using Independent Kruskal-Wallis Test and categorical variables compared using Pearson Chi-Square Test and Fisher’s Exact Test. a, b, c; sig at *p* < 0.05

**Fig. 2:**
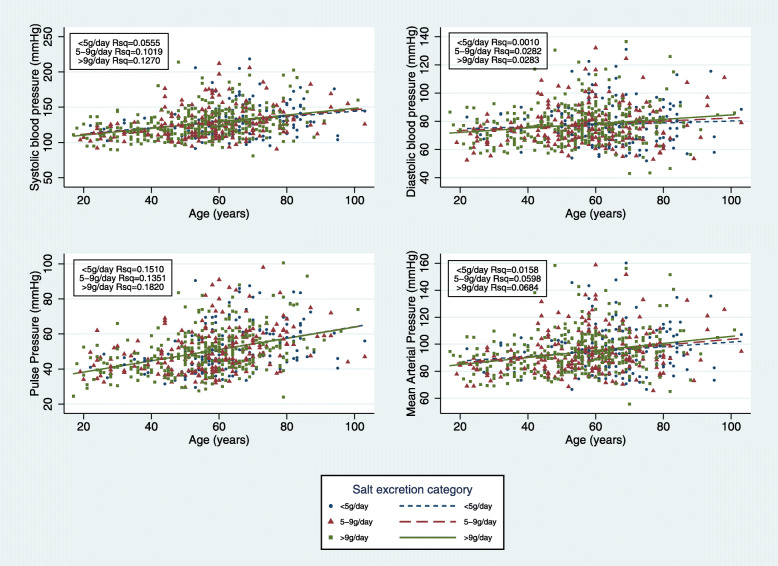
Slope of systolic, diastolic, pulse pressure and MAP with age in low (< 5 g/day, *n* = 154), medium (5–9 g/day, *n* = 220) and high (> 9 g/day, *n* = 302) salt groups excluding those on bp medication

**Fig. 3:**
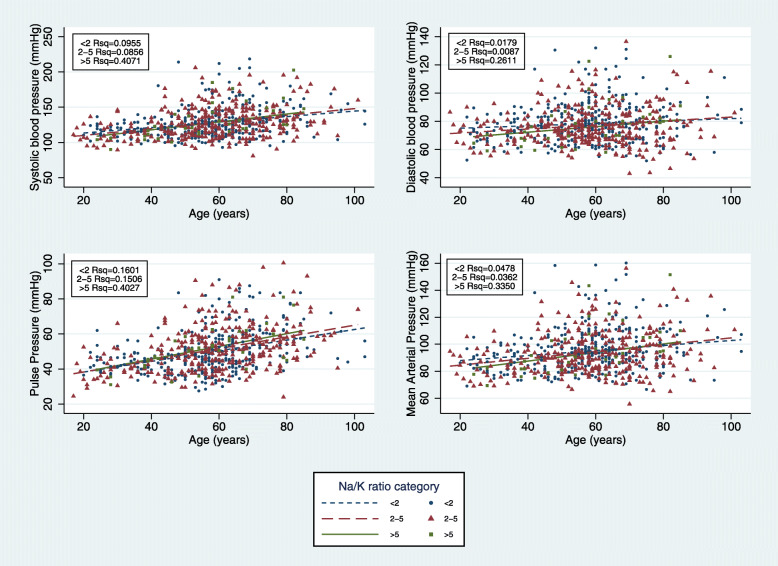
Slope of systolic, diastolic and pulse pressure with age in low (< 2 mmol/mmol, *n* = 334), medium (2–5 mmol/mmol, *n* = 308) and high (> 5 mmol/mmol, *n* = 34) Na: K groups excluding those on bp medication

Excluding all participants on treatment for hypertension (*n* = 154), a Spearman’s rank order correlation showed no association between Na excretion, K excretion or Na: K ratio and SBP, DBP or PP. We also found no significant difference between the regression slopes of SBP, DBP or PP with age in low (< 5 g/d, *n* = 154), medium (5-9 g/d, *n* = 220) and high (> 9 g/d, *n* = 302) salt groups (Fig. 2). However, there was an association between BP and the age regression slope according to urinary Na: K ratio. Compared with low Na: K ratio (< 2) and medium Na: K (2–5) ratios, the high Na: K group (> 5) had a significantly steeper slope with age for both SBP and DBP (*p* = 0.01 and *p* <  0.01 respectively). No significant differences for the slopes in all three Na: K groups was found for PP (Fig. 3). Among the high salt group (> 9 g/d), univariate analysis showed a significant relationship between age, father’s education, diabetes, K and physical activity with SBP while sex, age and father’s education were associated with DBP.

## Discussion

In a nationally representative sample of adult Ghanaians (50 years and older), we report that 24 h urinary Na excretion equated to a median salt intake of 8.3 g/day, with more than three-quarters of participants having salt intakes in excess of the WHO recommendation of < 5 g/day. This is considerably higher than median salt consumption values of 6.8 g and 7.2 g/day reported by recent studies from South Africa [31, 39], a country that has adopted mandatory salt reduction policies. Surprisingly, salt intake in the current study was higher in women than men, which is in contrast to findings of other studies [40, 41], and warranting further investigation.

Our data provides much-needed information required to inform strategies to meet the WHO global voluntary target for NCD prevention of 30% reduction in mean population intake of salt, with the aim of achieving a target of less than 5 g per day by 2025 [8]. The only other available information on 24 h urinary Na excretion in Ghana was collected over a decade ago when salt intake was estimated to be 6.0 g/day [42]. Over this time, the Ghanaian food environment has changed dramatically [43, 44] with the emergence of energy dense, nutrient poor, processed convenience foods such as instant noodles, salty snacks (eg, potato, corn, and tortilla chips), visible fat (e.g. butter, margarine, oils, dressings and gravies), ready-made baked foods (e.g. cookies, cakes, pies and pastries), desserts such as puddings and cheesecake, and sweetened products (e.g. sugar, syrup, ice cream, candy, and carbonated and noncarbonated sweetened drinks). Additionally, frequent consumption of highly salted foods, such as fish and meat, remains part of the traditional Ghanaian cuisine, and salt use in cooking remains high [42, 45].

It is noteworthy that more than three quarters of participants in the current study reported adding salt to food during cooking. Poor salt use behavior is widespread in the Ghanaian population; a previous study involving 12 villages in Ghana in 2006 reported that 98% of participants added salt to food during cooking, while about half (52%) added salt to food at the table [21]. In comparison to South Africans, Ghanaians reported adding salt to food during cooking more frequently [45], presumably because mass media public health campaigns to lower salt use, such as Salt Watch in South Africa have not yet occurred in Ghana [46]. Further, Ghana’s national salt iodization programme, along with accompanying major public health campaigns on iodine consumption [47–50] may partially be responsible for an increase in discretionary salt intake. However, lack of information on sources of salt provided from discretionary and non-discretionary sources, as in processed foods, prevents further hypothesis in this regard.

Despite the current study not including a dietary assessment component, a recent systematic review of dietary sources of salt in LMICs identified that bread, meat and meat products, bakery products, instant noodles, salted preserved foods, milk and dairy products, and condiments were major sources thereof [51]. In some LMICs, bread alone may contain as much as 1.36 g salt per 100 g bread [52]. For many African countries, bread has become a staple food, such as in some regions of Democratic Republic of Congo, where bread has replaced cassava [53]. The nutrition transition taking place in Sub Saharan Africa and its transformation of dietary patterns from a reliance on traditional staples to an increased intake of energy dense, nutrient poor foods (and often Na rich) foods may partly be responsible for the observed high salt intake reported by this study. In support of this, the Ghana Demographic and Health Survey 2014 reported that more than a third (36%) of participants consumed salted dried fish [54], while in the Ghana National Iodine Survey Report 2015, consumption of bouillon cubes was frequent and widespread with nearly half (48.8%) of the participants having consumed this item at least 6 times a week [55].

Awareness of excessive salt intake was low in the current study. Sixty one percent of participants believed that they were meeting the salt recommendation guidelines, but only 22.3% actually achieved this. Estimations of dietary salt intake using dietary recall methods usually indicates a reduced daily intake compared to 24 h urinary collections [56–58]. The tendency to under-estimate salt consumption is high as many people are unaware of the recommendation for salt consumption, unable to determine the salt content of everyday foods such as bread and cereals, or that of composite meals [59–61]. In Ghana, there are no salt reduction strategies in place such as: mandatory or voluntary levels of salt permitted in processed foods; regulations for food labelling to indicate salt content in products, or front-of-pack signposting for high salt warnings. In addition, the Ghanaian food composition tables are outdated and incomplete in the case of Na [62], making it difficult to assess food contributors to total salt intake. In view of this, regular monitoring and evaluation of the food environment and food consumption patterns are warranted.

Potassium excretion, although low (median 63.8 mmol /day, IQR 74.8, with 65% not meeting the recommended daily intake of K), was higher than that reported by other studies in the region [31, 39]. The K content of the food items obtained from the 2010–2011 Ghana Food Balance Sheet indicated that K supply per capita per day was about 9086 mg/day (233 mmol/day), approximately 2.6-fold larger than the WHO recommended level (> 90 mmol/day) with yams, cassava and plantains constituting the bulk of K supply [63, 64]. However, food balance sheets represent apparent, rather than actual food consumption, and thereby provide an overestimation of intake.

Hypertension prevalence in the current study was high (50 + y, 52.6%; 18-48y, 14.5%), with less than two thirds (60%) of participants classified as hypertensives being aware of the condition (i.e. diagnosed), of which 69.1% were being treated with antihypertensive medication, half of which had BP levels considered to be controlled (< 140/90 mmHg). Surprisingly, we found no association between urinary Na, K or N:K ratio with BP in contrasts to findings from other comparable studies [65–67] but in agreement with others [31, 68]. A possible reason may be the day to day variability in salt intake, which results in a high intra-individual variability in urinary Na excretion. Though 24 h Na excretion is considered to be the gold standard method for determination of population Na intake, repeated 24 h collections over periods provide more accurate estimating an individual’s habitual salt intake [69, 70]. However, this approach is typically impractical for large population studies such as the one reported here.

Similarly, no difference was found between the slopes of SBP, DBP or PP with age according to three categories of salt intake, namely low (5 g/day), medium (5-9 g/day) and high (> 9 g/day). However, high and medium urinary Na: K had steeper slopes of BP with age as compared with those in the low Na: K category. A national survey (WHO SAGE Wave 2 South Africa) conducted in 2015 reported similar findings [31]. This association was first explored in the INTERSALT study, the largest multinational study on 24 h urinary excretion and BP with 10,079 adults across 48 centres worldwide [66]. This implies that a simultaneous increase in K and a decrease in Na will be beneficial in reducing BP with age and ultimately hypertension incidence [38, 66]. As such, strategies such as taxes on high-salt products, labelling and effective communication, voluntary salt commitments by food industries and legislations for Na reduction are warranted. Similarly, to increase K intake, nutrition promotion of K rich fruits and vegetables may be needed, along with policies that make these foods more accessible and affordable.

A strength of the study is the use of a large nationally representative sample of participants aged 50+ years, in keeping with the aim of WHO SAGE studies. This design on the other hand, may limit generalization to the entire population. Another potential limitation is the recruitment strategy which resulted in more women than men. Women are known to be more likely to volunteer to participate in surveys than men [71], and this has been observed in other 24 h urine collection studies [31, 39]. Akan is the largest ethnic group in Ghana that is well distributed across the entire country [54] which is why this group was proportionally larger than other ethnic groups in the sample. Additionally, the cross-sectional analysis limits the ability to evaluate the influence of dietary Na and K intake on BP over time. The choice of urine volumes as a sole determinant of completeness of 24 h urine collections requires explanation. Presently, there is no universally accepted standard for determining completeness of 24 h urine collections. The International Consortium for Quality Research on Dietary Sodium/Salt (TRUE), recommends the use of para-aminobenzoic acid (PABA) as being the preferred method [72] but experiences using PABA in African communities is limited and experiences from South Africa [73] suggest that participants may forget or choose not to take their PABA tablets during the urine collection period, thus rendering this method impractical .

The TRUE consortium recognized a need to establish further additional measures of assessing 24 h urinary completeness beyond PABA. A systemic review found that no single exclusion criteria (24 h urinary creatinine excretion, creatinine index < 0.7, total urinary volume, a combination of creatinine excretion and total urine volume or self-report of missing urine) appeared more accurate in identifying incomplete 24-h urine collections than any others [74]. Importantly, the authors point out that the use of a combination of methods such as creatinine excretion together with urinary volume may lead to the unnecessary exclusion of participants with complete urine samples. The reference values used as a cut-off for inadequate urinary collection based on creatinine values has largely been based on those recommended by Stolarz-Skrzypek et al., namely urinary volume < 300 ml, or creatinine excretion < 4 mmol or > 25 mmol for women and < 6 mmol and > 30 mmol for men [28]. The applicability of these creatinine excretion reference values across different ethnic groups has recently been questioned [74]. While the TRUE authors concluded that a creatinine index < 0.7 may best increase the sensitivity of eliminating incomplete urine sampling, they go on to promote the use of similar methodologies used by the INTERSALT and INTERMAP studies as the preferred method to determine 24 h urinary completeness in population based studies [27, 75]. These were as follows: samples that fell outside of the collection time of 22–26 h; participants’ indication that the collection was incomplete and that they had spilled ‘more than a few drops’ of urine; or if total urinary volume was < 250 ml. Given that creatinine is generally variable and largely dependent on muscle mass and protein intake [74], and the reference cut-off values in African populations have not been widely validated, we opted to use 24 h urinary volumes ≤300 ml as the measure for incomplete collections. The generally low creatinine excretion values found across the entire cohort suggest a possible low dietary protein intake, but a lack of dietary data precludes further consideration in this regard.

## Conclusions

This nationwide study of Ghanaians, using 24 h urinary collections, reported a high intake of salt, accompanied by a low K intake. Increasing urinary Na: K ratio was associated with increasing BP with age. Given the high prevalence of hypertension in the study, our findings identify a need for population-wide strategies to reduce dietary salt intake whilst, at the same time, increase K intake in Ghana.

## Supplementary information

**Additional file 1: Table S1.** Na, K, Cl and Cr of original and repeat data in the same participants in Alajo EA. **Table S2.** Urine results for electrolytes, creatinine and iodine by sex (urine data considered valid if volume > 300 ml), WHO-SAGE Ghana Wave 3. **Table S3.** Urine results for electrolytes, creatinine and iodine by age (urine data considered valid if volume > 300 ml), WHO-SAGE Ghana Wave 3. **Table S4.** Salt knowledge, attitudes and behaviour and fruits and vegetable consumption by age category, sex and location (*n* = 837), nested sub study; WHO-SAGE Ghana Wave 3.

## Data Availability

The datasets used and/or analyzed during the current study are available from the corresponding author on reasonable request.

## Abbreviations

CVDs: Cardiovascular diseases
Na: Sodium
K: Potassium
Cr: Creatinine
LMICs: Low- and Middle-Income Countries
NCDs: Non communicable diseases
WHO: World Health Organization
SHAKE: Surveillance, Harness industry, Adopt Standards for marketing and labelling, Knowledge and Environment
BP: Blood Pressure
SAGE: Study on global AGEing and adult health
CAPI: Computer Assisted Personal Interview
PAHO: Pan American Health Organization
NMIMR-UG: Noguchi Memorial Institute for Medical Research of the University of the Ghana
INTERMAP: International Population Study on Macronutrients and Blood Pressure
INTERSALT: International Study of Sodium, Potassium, and Blood Pressure
EAs: Enumerator Areas
SBP: Systolic Blood Pressure
DPB: Diastolic Blood Pressure
PP: Pulse Pressure
MAP: Mean Arterial Pressure
IQR: Inter Quartile Range
BMI: Body Mass Index
TRUE: The International Consortium for Quality Research on Dietary Sodium/Salt
PABA: Para-aminobenzoic acid
