# Supplementary material for: Salt and potassium intake among adult Ghanaians: WHO-SAGE Ghana Wave 3
Source: BMC Nutr. 2020 Sep 29;6:54. doi: 10.1186/s40795-020-00379-y (PMC7523323; doi:10.1186/s40795-020-00379-y)
Supplement: Supplementary file 1 — Additional file 1: Table S1. Na, K, Cl and Cr of original and repeat data in the same participants in Alajo EA. Table S2. Urine results for electrolytes, creatinine and iodine by sex (urine data considered valid if volume > 300 ml), WHO-SAGE Ghana Wave 3. Table S3. Urine results for electrolytes, creatinine and iodine by age (urine data considered valid if volume > 300 ml), WHO-SAGE Ghana Wave 3. Table S4. Salt knowledge, attitudes and behaviour and fruits and vegetable consumption by age category, sex and location (n = 837), nested sub study; WHO-SAGE Ghana Wave 3. [file 40795_2020_379_MOESM1_ESM.docx]

**Supplementary Table 1:** **Na, K, Cl and Cr of original and repeat data in the same participants in Alajo EA**

|  | Na (mmol/l) | | K (mmol/l) | | Cl (mmol/l) | | Creatinine (µmol/l) | |
| --- | --- | --- | --- | --- | --- | --- | --- | --- |
| Statitsics | Original data | Repeat data | Original data | Repeat data | Original data | Repeat data | Original data | Repeat data |
| Minimum | 64.40 | 65.45 | 10.70 | 25.20 | 61.40 | 37.95 | 0.68 | 1.51 |
| Median | 249.40 | 126.33 | 78.85 | 65.43 | 269.40 | 93.85 | 1.94 | 4.24 |
| Maximum | 741.20 | 250.45 | 356.00 | 141.20 | 951.60 | 206.80 | 5.91 | 15.03 |
| Mean | 311.69 | 135.63 | 108.23 | 67.39 | 355.73 | 110.20 | 2.38 | 4.61 |
| Sd | 238.20 | 53.01 | 93.32 | 30.94 | 271.09 | 47.95 | 1.61 | 3.11 |
| N | 16 | 16 | 16 | 16 | 16 | 16 | 16 | 16 |

**Supplementary Table 2. Urine results for electrolytes, creatinine and iodine by sex (****urine data considered valid if volume > 300ml),** **WHO**-**SAGE Ghana Wave 3**

|  | All  (n = 835) | Men  (n =269) | Women  (n = 566) | P |
| --- | --- | --- | --- | --- |
| *24hour Urine Analysis* |  |  |  |  |
| Sodium, mmol/24hr | n = 829  142.3 (130.8) | n = 267  126.6 (125.7) | n = 562  146.2 (127.3) | 0.01 |
| Salt excretion, g/day | n = 829  8.3 (7.5) | n = 267  7.5 (7.4) | n = 562  8.7 (7.5) | 0.01 |
| Achieving salt target (<5g/day), n (%) | n = 836  203 (24.3) | n = 276  72 (27) | n = 560  108 (19.3) | 0.01 |
| Potassium (K), mmol/24hr | n = 835  65.9 (79.5) | n = 269  62.6 (72.4) | n = 566  65.8 (77.4) | 0.38 |
| Achieving K target (≥90 mmol/day), n (%) | n = 835  357 (42.7) | n = 269  91 (33.8) | n = 566  200 (35.3) | 0.67 |
| Sodium-to-potassium ratio | n = 829  2.1 (1.7) | n = 267  1.9 (1.5) | n = 562  2.1 (1.7) | 0.06 |
| Achieving Na:K ratio (≤1.0), n (%) | n = 825  59 (6.3) | n = 267  21 (7.9) | n = 558  31 (5.6) | 0.20 |
| Cr, mmol/24h | n = 835  3.4 (2.4) | n = 269  3.6 (2.6) | n = 566  3.3 (2.3) | 0.15 |
| 24hr Iodine, µg/l | n = 799  140.8 (140.1) | n = 255  154.6 (152.3) | n = 544  137.8 (137.8) | 0.02 |

Data are presented as median (IQR) unless otherwise stated; Mann-Whitney Test used to compare medians, Pearson Chi-Square test and Fisher’s Exact Test used to compare proportional data.

**Supplementary Table 3: Urine results for electrolytes, creatinine and iodine by age (urine data considered valid if volume > 300ml),** **WHO-SAGE Ghana Wave 3**

|  | ≥50 years  (n = 661) | 18-49yrs  (n = 174) | P |
| --- | --- | --- | --- |
| *24hour Urine Analysis* |  |  |  |
| Sodium, mmol/24hr | n = 656  136.4 (120.3) | n = 173  164.3 (138.2) | < 0.01 |
| Salt excretion, g/day | n = 656  8.1 (7.1) | n = 173  9.7 (8.2) | < 0.01 |
| Achieving salt target (<5g/day), n (%) | n = 654  159 (24.3) | n = 173  21 (12.1) | < 0.01 |
| Potassium (K), mmol/24hr | n = 661  61.7 (66.8) | n = 174  80.4 (93.3) | < 0.01 |
| Achieving K target (≥90 mmol/day), n (%) | n = 661  212 (32.1) | n = 174  79 (45.4) | < 0.01 |
| Sodium-to-potassium ratio | n = 656  2.1 (1.6) | n = 173  2.1 (1.8) | 0.33 |
| Achieving Na:K ratio (≤1.0), n (%) | n = 653  39 (6.0) | n = 172  13 (7.6) | 0.45 |
| Cr, mmol/24h | n = 661  3.4 (2.2) | n = 174  4.1 (2.5) | < 0.01 |
| 24hr Iodine, µg/l | n = 629  144.2 (141.6) | n = 170  146.5 (137.5) | 0.63 |

Data are presented as median (IQR) unless otherwise stated; Mann-Whitney Test used to compare medians, Pearson Chi-Square test and Fisher’s Exact Test used to compare proportional data.

**Supplementary table 4: Salt knowledge, attitudes and behaviour and fruits and vegetable consumption by age category, sex and location (n = 837), nested sub study; WHO-SAGE Ghana Wave 3.**

|  | All  (n = 837) | Age Category  (n = 837) | | p value | Sex (837) | | p value | Location (837) | | p value |
| --- | --- | --- | --- | --- | --- | --- | --- | --- | --- | --- |
|  |  | 50+ yrs | 18-49 yrs |  | Men | Women |  | Urban | Rural |  |
| Add salt to food at the table, frequently n (%) | 107 (12.8) | n = 664  78 (11.7) | n = 173  28 (27.3) | 0.02 | n = 270  39 (14.4) | n = 568  68 (12.0) | 0.41 | n = 456  46 (10.1) | n = 381  61 (16.0) | <0.01 |
| Add salt to cooking at home, frequently n (%) | 671 (80.1) | n = 664  520 (78.3) | n = 173  150 (86.7) | 0.03 | n = 270  219 (81.1) | n = 568  452 (79.6) | 0.69 | n = 456  358 (78.5) | n = 381  312 (81.9) | 0.24 |
| How much salt consumed, just the right amount n (%) | 508 (60.9) | n = 662  387 (58.5) | n = 171  120 (70.2) | < 0.01 | n = 269 178 (66.2) | n = 565  330 (58.4) | 0.10 | n = 455  265 (58.2) | n = 378  242 (64.0) | 0.09 |
| Can high salt diet cause a serious health problem, yes no (%) | 651 (77.8) | n = 643  517 (80.5) | n = 170  134 (78.8) | 0.65 | n = 264  216 (81.8) | n = 549  435 (79.2) | 0.39 | n = 444  362 (81.5) | n = 368  288 (78.3) | 0.25 |
| Do you do anything on regular basis to control salt consumption, no (%) | 394 (51.5) | n = 643  320 (49.8) | n = 169  74 (43.8) | 0.17 | n = 259  136 (52.5) | n = 553  282 (51.0) | 0.69 | n = 445  216 (48.5) | n = 366  201 (54.9) | 0.72 |
| Fruits and vegetable intake, did not meet recommendation  n (%) | 396 (47.2) | n = 644  312 (47.0) | n = 173  83 (48.0) | 0.82 | n = 270  126 (46.7) | n = 568  270 (47.5) | 0.81 | n = 457  209 (45.7) | n = 381  186 (48.8) | 0.37 |

Data was recorded in frequencies. Chi square tests were conducted.
